# Cooperation of decay-accelerating factor and membrane cofactor protein in regulating survival of human cervical cancer cells

**DOI:** 10.1186/1471-2407-9-384

**Published:** 2009-10-30

**Authors:** Ling-Juan Gao, Shu-Yu Guo, You-Qun Cai, Ping-Qing Gu, Ya-Juan Su, Hui Gong, Yun Liu, Chen Chen

**Affiliations:** 1 Clinical Laboratory, Nanjing Maternity and Child Health Care Hospital, Nanjing, PR China; 2 Clinical Laboratory, Southern Medical University, Guangzhou, PR China

## Abstract

**Background:**

Decay-accelerating factor (DAF) and membrane cofactor protein (MCP) are the key molecules involved in cell protection against autologus complement, which restricts the action of complement at critical stages of the cascade reaction. The cooperative effect of DAF and MCP on the survival of human cervical cancer cell (ME180) has not been demonstrated.

**Methods:**

In this study we applied, for the first time, short hairpin RNA (shRNA) to knock down the expression of the DAF and MCP with the aim of exploiting complement more effectively for tumor cell damage. Meanwhile, we investigated the cooperative effects of DAF and MCP on the viability and migration, moreover the proliferation of ME180 cell.

**Results:**

The results showed that shRNA inhibition of DAF and MCP expression enhanced complement-dependent cytolysis (CDC) up to 39% for MCP and up to 36% for DAF, and the combined inhibition of both regulators yielded further additive effects in ME180 cells. Thus, the activities of DAF and MCP, when present together, are greater than the sum of the two protein individually.

**Conclusion:**

These data indicated that combined DAF and MCP shRNA described in this study may offer an additional alternative to improve the efficacy of antibody-and complement-based cancer immunotherapy.

## Background

Cervical cancer is the second most common cancer among women worldwide, with about 470,000 newly diagnosed cases and almost 250,000 deaths every year [1,2]. Cervical cancer is the leading cause of death from cancer in many low-resource countries [3]. The lack of preventive strategies, early diagnostic methods, and effective therapies to treat recurrent cervical tumors creates a pressing need to understand its pathogenesis and to identify molecular markers and targets for diagnosis as well as therapy [4]. The unlimited growth and the metastasis are the important traits of the tumor, are the main causes of cancer-related death induced by the failure of treatment of cervical cancer [5]. However, the factors that promote malignant transformation and growth in cervical carcinoma remain largely unknown.

The complement system has been characterised extensively, both biochemically and functionally. When complement components are deposited on host cells, complement regulating factors protect autologous cells from complement mediated cytotoxicity [6]. Antibody-mediated complement-dependent killing of tumor cells is not a very efficient effector mechanism, due to the overexpression of complement regulatory proteins on tumor cells, which are protected from complement attack in this way. Decay-accelerating factor (DAF) and membrane cofactor protein (MCP) function as cell surface regulators that serve to protect self cells from attack by autologous complement. The two proteins complement each other in that DAF acts to accelerate the decay of the classical and alternative C3 convertases (C4b2a and C3bBb) [7] while MCP functions as a cofactor for the factor I-mediated cleavage of cell-bound C3b and C4b [8]. Although both proteins have been studied extensively, little is known about whether and, if so, how they cooperate on cervical cancer cells surface. Therefore, our present study aimed to investigate the expression of DAF, MCP and to assess effect of the protein on cervical cancer cells survival.

## Methods

### Reagents

Human cervical cancer cell lines ME180 (The level of DAF and MCP in ME180 was the maximal expression [see Additional file 1]) was provided by China Centre for Type Culture Collection. The short hairpin RNA (shRNA) was synthesized by Wuhan Genesil Biotechnology Co., Ltd (Wuhan, China). Lipofectamine 2000 was purchased from Invitrogen (Carlsbad, CA). Phototope-HRP Western Blot Detection System, including anti-mouse IgG, HRP-linked antibody, Biotinylated protein ladder, 20× LumiGLO Reagent and 20× Peroxide were purchased from Cell Signaling Technology (Beverly, MA, USA). The mouse monoclonal antibodies against decay-accelerating factor (DAF), membrane cofactor protein (MCP) and β-actin were purchased from Santa Cruz Biotechonolgy (Santa Cruz, CA, USA). The ^3^H-thymidine was endowed by isotope lab of Nanjing Medical University. Trizol reagent was purchased from life Techology (Gaitherburg, MD, USA). Human blood was obtained from healthy donors.

### Tissue procurement and preparation

Human cervical cancer tissues were collected from 30 patients who underwent radical hysterectomy because of cervical carcinoma at Nanjing Maternity and Child Health Care Hospital between October 2004 and January 2007. Tumor specimens were obtained immediately after surgery. Local ethical approval was obtained before commencing this study and, as appropriate, tissue was collected with informed consent.

### Human cervical cancer cell line culture and DNA transfection

The human cervical cancer cells were propagated in Dulbecco's modified Eagle's medium (DMEM) supplemented with 10% Fatal Calf Serum (FCS), 100 U/ml penicillin, 100 μg/ml streptomycin, 10% unnecessary amino acid at 37°C in a 5% CO_2 _incubator. Cells used in experiments were from 5 to 7 passages. Lipofectin- and oligofectamine-transfection of shRNA was performed according to the manufacturer's recommendations. Briefly, to transfect tumor cells of one well of a six-well plate, each 500 pmol shRNA and 10 μl lipofectin were diluted in 750 μl OptiMEM. After preincubation of the lipofectin solution for 45 min at 37°C, both solution were mixed and incubated for additional 15 min at room temperature. The lipofectin/shRNA mixture was subsequently overlaid onto the cells and incubated for 2 h. Finally, 1 ml growth medium (20% FCS) per well was added for further cultivation of the tumor cells. Reporter gene activities were normalized to total protein, and all results represented the average of triplicate experiments.

### Construction of DAF shRNA-expressing plasmid vector

The complementary oligonucleotides encoded a hairpin structure with a 19-mer stem derived from the target site, in this experiment, the targeted short hairpin RNA (shRNA) sequences for DAF were CTC CAC TGG ACA GAG CTG CC and MCP were GCG CGG CGC GGA AGA CGC TG. Two complementary domains were separated by a 9-bp loop sequence. Near the 3' end of the shRNA template was a 6 nucleotide poly (T) tract recognized as a RNA pol III termination signal. The 5' end of the two oligonucleotides was BamHI and Hind III restriction site overhung. The vectors of DAF and MCP shRNA-expressing plasmid were constructed by using pGenesil-1 as the vector backbone. The shRNA synthesized and annealed was ligated into the BamHI and Hind III site of the pGenesil-1 expression vector. At the same time, we chose an unrelated gene shRNA as a negative control.

### Western blot analysis

The cells were collected with sample buffer. Whole cell lysates (50 μg) for each sample were subjected to electrophoresis in 10% SDS-polyacrylamide gels. Thereafter, the protein was blotted onto a PVDF membrane. Primary antibodies against DAF, MCP and β-actin were used according to the manufacturer's recommedations. After washing the membrane, the second antibody (HRP-conjugated anti-mouse IgG) was used for the detection of DAF, MCP and β-actin. The bands were visualized by the ECL detection system with 5 to 10 min exposure after washing the membrane. β-actin was used as a protein loading control.

### C3-deposition

ME180 cells were incubated and subsequently sensitized by incubation with a rabbit polyclonal anti-ME180 antiserum (diluted 1/5) for 20 min on ice. ME180 cells were washed, resuspended at 3 × 10^6 ^cells/ml and 50 μl cells were mixed with 100 μl of C8-depleted serum (prepared by passage over an anti-C8 affinity column). After 1 hr incubation at 37°, complement activation was terminated by washing cells once with ice-cold EDTA solution (20 mM EDTA/FACS buffer) and two additional times with FACS buffer. Flow cytofluorometrical analysis, as described above, was used to quantify C3b binding.

### Complement-mediated cytotoxicity

A radioactive cytotoxicity assay was used to measure complement-mediated cytotoxicity. ME180 cell transfectants were grown to 70% confluency on 100-mm culture plates. Cells were removed with 4 ml Versene and washed three times with PBS. A total of 1 × 10^7 ^cells then were suspended in 1 ml PBS containing 500 μCi ^51^Cr, and the cell suspensions were incubated for 2 h at 37°C with occasional shaking. After washing three times with GVB-E, 5 × 10^5 ^labeled cells were incubated for 15 min at 4°C with rabbit anti-hamster lymphocyte serum (1:2) in 200 μl of GVB-E. Cells were washed three more times with GVB^2+^, resuspended to 10^5 ^cells/ml in GVB^2+^, and 100-μl aliquots of the cell suspension added to the wells of 96-well V-bottom plates. Then serial dilutions of normal human serum were added in 100-μl volumes in triplicate wells for each cell type. A volume of 100 μl of 1% Triton X-100 and buffer alone were included as controls for 100% release and for spontaneous release, respectively. The percent specific release was calculated from the formula: % specific release = [(measured release - spontaneous release)/(100% release - spontaneous release)].

### Cell viability assay

We confirmed proliferating activity by water-soluble tetrazolium salt (WST-1) assay (Roche Diagnostics, Mannheim, Germany). The WST-1 assay is a colorimetric method in which the dye intensity is proportional to the number of viable cells. Cells were seeded into 96-well microtiter plates at a concentration of 5 × 10^3 ^cells/well. After 12-h incubation, cells were treated with different media for 48 h. After incubation, the cells were washed with PBS and the cell proliferation reagent WST-1 was added, then incubated for 4 h. Sample absorbence was analyzed with a bichromatic ELISA reader at 450 nm. All experiments were performed in triplicate with different passages of the ME180 cells.

### In vitro migration assay

Cell migration was assayed using 24-mm diameter chambers with 8-μm pore filters (Transwell, 6-well cell culture). The ME180 cells were removed from the culture flasks and resuspended at 7.5 × 10^6 ^cells/ml in serum-free medium, and then 0.2 ml cell suspension was added to the upper chambers. Afterwards, the lower chambers were added with the different medium (0.5 ml). The chambers were incubated for 48 hours at 37°C in a humid atmosphere of 5% CO2/95% air. And then the filters were fixed in 95% ethanol and stained with H.E. The upper surfaces of the filters were scraped twice with cotton swabs to remove non-migrated cells. The experiments were repeated in triplicate with different passages of the ME180 cells, and the migrated cells were counted microscopically (400×) in five different fields per filter.

### Measurement of ^3^H-thymidine incorporation (^3^H-TdR)

ME180 were plated into 96-well plates and incubated overnight. Media were removed from the cells and replaced with 200 μl media, as described in the results section. The cells were incubated for 54 h, and then DNA synthesis was determined by ^3^H-TdR for the final 18 h. The media were carefully removed and the cells were detached with 50 μl trypsin-EDTA. The cells were then harvested onto glass filters with a Tomtech cell harvester and the radioactivity retained on the dried filters was measured by the addition of 50 ml scintillation liquid and counted in a TopCount NxT scintillation counter. All experiments were performed in triplicate with different passages of the ME180 cells.

### Statistical analysis

Most results are presented as means ± SD. Differences between various data sets were tested for significance using Student's t-test and p-value of less than 0.05 were considered significant (*p < 0.05; **p < 0.01; ***p < 0.001).

## Results

### The complement regulatory protein expression and C3b deposition in human cervical cancer tissue

**Figure 1:**
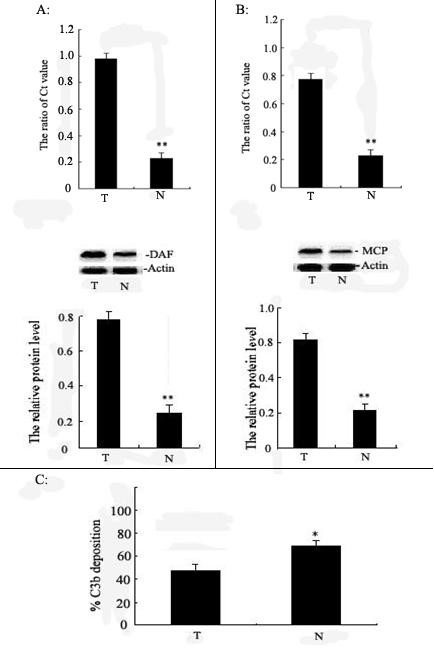
**The DAF and MCP expression levels and C3b deposition in cancer tissue**. **A**: Relative DAF expression levels were shown between human cervical cancer tissues (T) and surrounding non-neoplastic tissues (N). The expression of DAF mRNA and protein was measured by Real-time PCR and Western blot respectively, The DAF gene in human cervical cancer tissue was overexpressed. Results shown are the mean ± SD of three independent transfections (n = 3), each conducted in triplicate. **B**: Relative MCP expression levels were shown between human cervical cancer tissues (T) and surrounding non-neoplastic tissues (N). The expression of MCP mRNA and protein was measured by Real-time PCR and Western blot respectively, The MCP gene in human cervical cancer tissue was overexpressed. Results shown are the mean ± SD of three independent transfections (n = 3), each conducted in triplicate. **C**: Deposition of opsonizing C3 split products were shown between human cervical cancer tissues and surrounding non-neoplastic tissues (measured as common C3b moiety). Data are presented as means ± SD of two independent experiments. Student's t-test: N versus T. **p < 0.01; *p < 0.05, respectively.

In order to investigate the relationship between complement regulatory protein and complement-mediated cytotoxicity in human cervical cancer cells, the DAF, MCP expression and C3b deposition of 30 cases human cervical cancer tissues and surrounding non-neoplastic tissues were analyzed in this experiment (Figure 1). The expression of DAF and MCP was significantly increased in human cervical cancer tissue compared with surrounding non-neoplastic tissues. Meanwhile, a further significant decrease of C3b deposition could be demonstrated in human cervical cancer tissues. This finding suggested that DAF and MCP may play an important role in survival of human cervical cancer cells.

### MCP and DAF work synergistically in preventing C3b deposition

**Figure 2:**
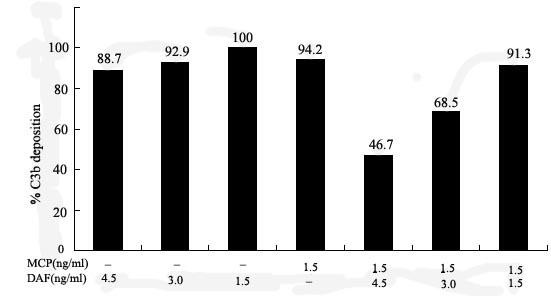
**C3b deposition**. Cooperative inhibition of C3b deposition by DAF and MCP was shown in this experiment. At the low levels tested, DAF had only minor effects on C3b deposition when present alone. However, when present with low levels of MCP, the inhibition dramatically increased. For each experiment, the data given are the mean ± SD, where n = 3.

In this experiment, MCP and DAF protein were studied together. As shown in Figure 2, MCP alone on the cell surface conferred minimal inhibition of C3b deposition(5.8%), as did a range of limiting DAF concentrations(0-11.3% inhibition). In contrast, inhibition increased dramatically when both proteins were incorporated into the cells (up to 53.3%). With the fixed limiting dose of MCP, inhibition was again dependent on the concentration of the DAF added.

### Functional analysis of MCP and/or DAF shRNA-mediated inhibition of MCP and/or DAF expression

**Figure 3:**
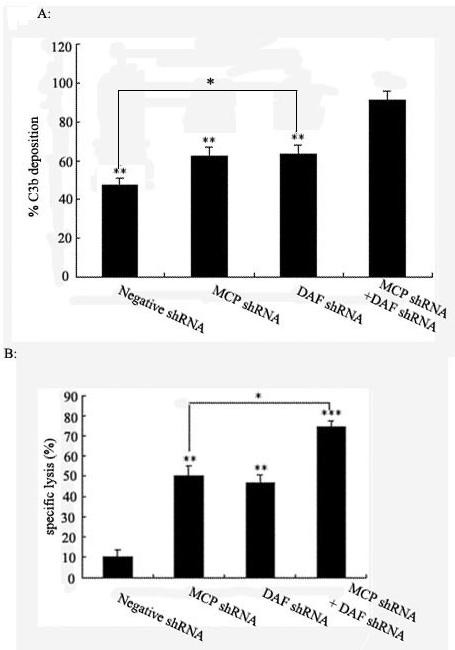
**C3b deposition and complement-mediated cytotoxicity**. **A**: Deposition of opsonizing C3 split products on DAF- and/or MCP-deficient tumour cells (measured as common C3b moiety). Tumor cells were transfected with negative shRNA, MCP shRNA, DAF shRNA and MCP shRNA+ DAF shRNA respectively, as indicated in the diagram. Following complement activation by polyclonal tumor-specific antibodies, deposited C3b molecules were quantified by flow cytometry. Data are shown from one representative experiment (of three). Negative shRNA, MCP shRNA, DAF shRNA versus MCP shRNA+ DAF shRNA. **p < 0.01; MCP shRNA, DAF shRNA versus negative shRNA. *p < 0.05, respectively. **B**: Complement-dependent cytolysis of DAF- and MCP-deficient ME180 cells. Tumor cells were transfected with negative shRNA, MCP shRNA, DAF shRNA and MCP shRNA+ DAF shRNA respectively, as indicated in the diagram. 72 h later, complement-dependent cytolysis of ME180 cells was measured. Data are presented as means ± SD of triplicates of one representative experiment (of three). DAF shRNA, MCP shRNA, MCP shRNA+ DAF shRNA versus negative shRNA. **p < 0.01; ***p < 0.001; MCP shRNA, DAF shRNA versus MCP shRNA+ DAF shRNA.*p < 0.05, respectively.

Because MCP and DAF are regulators of the early complement pathway, their knock-down was also expected to improve C3 opsonization of tumor cells. Therefore, C3 split product deposition (measured as C3b) was analysed on MCP and/or DAF-deficient tumor cells following complement activation. Tumor cells were transfected with negative shRNA, MCP shRNA, DAF shRNA and MCP shRNA+ DAF shRNA respectively. The results showed improved C3b opsonization upon MCP and DAF suppression, where down-regulation of MCP and DAF did enhance complement-mediated cytotoxicity significantly (Figure 3A).

A further significant augmentation of complement-mediated cytotoxicity could be demonstrated in ME180 cells upon simultaneous transfection of MCP shRNA+ DAF shRNA. Compared to complement-mediated cytotoxicity following knock-down of MCP (most potent single effect), a combined inhibition of MCP and DAF expression further enhanced significantly complement-mediated cytotoxicity of ME180 cells by 25%(Figure 3B)

### The effect of human cervical cancer cells viability, migration and proliferation induced by down-expression of MCP and/or DAF

**Figure 4:**
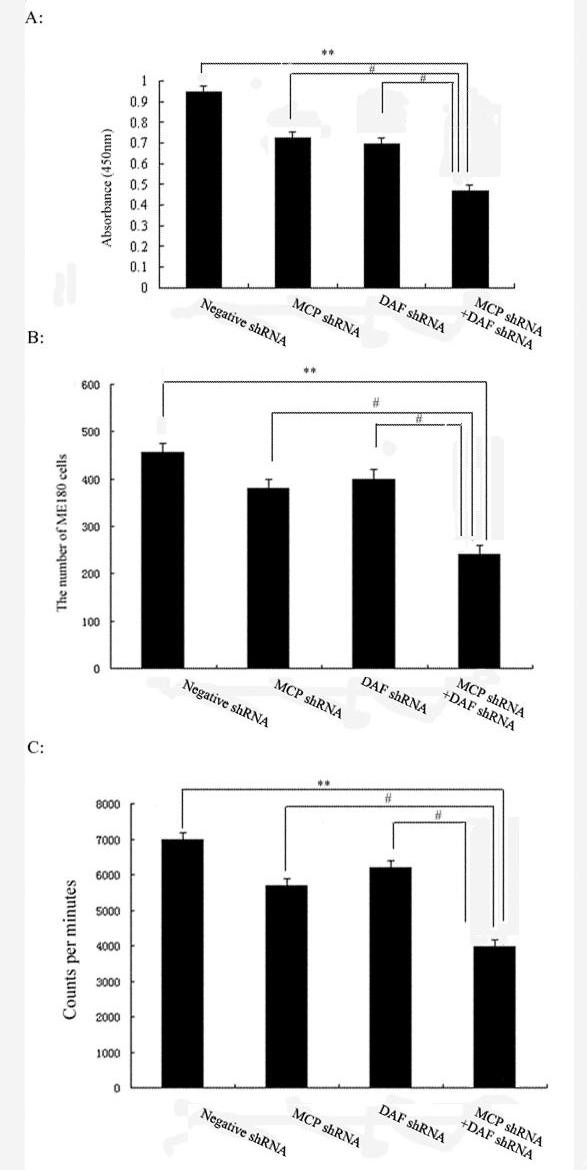
**Human cervical cancer cells viability, migration and proliferation**. ME180 cells (3 × 10^4^/ml) were treated with negative shRNA, MCP shRNA, DAF shRNA and MCP shRNA+ DAF shRNA respectively in this experiment. **(A)**: The ME180 cells viability was detected via WST-1 assay. Sample absorbance was analyzed using a bichromatic ELISA reader at 450 nm. **p < 0.01 versus MCP shRNA+ DAF shRNA, ^#^*p *< 0.05 versus MCP shRNA+ DAF shRNA. (B): The migration of ME180 cells was measured by Transwell assay. The migrated cells were counted microscopically (400×) in five different fields per filter. **p < 0.01 versus MCP shRNA+ DAF shRNA, ^#^*p *< 0.05 versus MCP shRNA+ DAF shRNA. **(C) **to observe the measuring ^3^H-thymidine incorporated into DNA over the last 18 h of the final incubation. Results were mean ± SD from 3 independent experiments. **p < 0.01 versus MCP shRNA+ DAF shRNA, ^#^*p *< 0.05 versus MCP shRNA+ DAF shRNA.

To investigate the effect of DAF and MCP on human cervical cancer cells viability, migration and proliferation, ME180 was transfected with negative shRNA, MCP shRNA, DAF shRNA and MCP shRNA+ DAF shRNA respectively. Cell viability was determined by WST-1 assay. As shown in Figure 4A, MCP shRNA+ DAF shRNA can significantly decrease cell viability, compared with negative shRNA, meanwhile, the viability of cells in MCP shRNA group and DAF shRNA group had slight change compared with MCP shRNA+ DAF shRNA group. The results indicated that cooperative inhibition of DAF and MCP genes resulted in dramatically decreasing viability of ME180 cells.

To determine whether DAF and MCP involved in regulation of cell migration, ME180 was transfected with negative shRNA, MCP shRNA, DAF shRNA and MCP shRNA+ DAF shRNA respectively. The number of migrated cell treated with MCP shRNA+ DAF shRNA was significantly lower than that in negative shRNA (*p *< 0.01). The silence of DAF and MCP synergistically can significant decrease the migration of ME180 cells. The numbers of migrated cells were also differences in MCP shRNA and DAF shRNA group when compared with MCP shRNA+ DAF shRNA group (*p *< 0.05), which indicated enhancing effect of DAF and MCP on cell migration.

As shown in Figure 4C, the exposure of MCP shRNA+ DAF shRNA decreased the proliferation of cervical cancer cell. There was an apparent decrease in ME180 DNA synthesis, exposed to MCP shRNA+ DAF shRNA for 24 h after the initial manipulation. The effect of negative shRNA, MCP shRNA and DAF shRNA exposure on cell proliferation was also determined. MCP shRNA+ DAF shRNA can significantly decrease the proliferation of cervical cancer cell, as compared with negative shRNA, MCP shRNA and DAF shRNA group respectively. As above, MCP and DAF can dramatically inhibit the proliferation of cervical cancer cell in a cooperative fashion.

## Discussion

The membrane attack complex inhibitory protein DAF and MCP are key members of the family of complement regulatory proteins. Due to the expression of membrane-bound complement regulatory proteins, complement deposition on neoplastic cells is limited and therefore not sufficient to induce potent tumour cell killing [9]. Silencing of complement regulatory proteins has been shown to sensitize tumour cells to complement attack [10]. Therefore, we applied a shRNA strategy to inhibit specifically the expression of DAF and MCP aiming at better employment of complement for tumour cell destruction.

Numerous studies have been performed on the complement regulatory proteins in primary tumors and in tumor cell lines, in an attempt to clarify their significance to cancer immunoresistance. The fact that most cancers, independent of their tissue origin, express at least two if not three complement regulatory proteins, is perhaps not surprising considering the wide tissue distribution of DAF and MCP [11]. Li et al [12] examined colorectal and gastric carcinomas and osteosarcoma and found increased expression of DAF, whereas Kiso et al [13] found increased expression of both DAF and MCP in intestinal type gastric carcinoma. In ovarian cancer, DAF and MCP were more heterogeneously expressed and resistance to complement correlated in these cells with high level of DAF and MCP expression [14]. In this study, we detected the expression of DAF and MCP protein in cervical cancer tissue by Western blot. Increasing changes in DAF and MCP protein were observed, the expression of DAF and MCP were significantly increased in human cervical cancer tissue compared with surrounding non-neoplastic tissues, Meanwhile, a further significant decrease of C3b deposition could be demonstrated in human cervical cancer tissues. This finding suggested that DAF and MCP may play an important role in survival of human cervical cancer cells.

Although DAF and MCP each have been studied extensively, no investigations have focused on whether the two proteins interact in providing optimal protection of self cells from autologous complement despite the fact that nearly all cells express both proteins. Using the above experimental system with incorporated DAF and MCP in ME180 cells, we demonstrated that the two regulators work synergistically on the cell surface in preventing alternative pathway-mediated C3b deposition. The magnitude of the inhibition of C3b uptake in the presence of the two proteins compared to each protein individually was striking. At higher concentrations of the proteins, this cooperative inhibition reached 53-72% as compared to 0-11% for each protein when the proteins were incorporated individually at the same concentrations.

Functional studies were performed to evaluate complement resistance of tumour cells after shRNA-mediated knock-down of DAF and/or MCP expression. The most striking effects were found that CDC was increased about 67% after inhibition of DAF and MCP; 39% and 36% increased CDC were apparent in MCP or DAF-deficient ME180 cells respectively. In contrast to the DAF and MCP shRNA approach, silencing DAF or MCP by shRNA had only a weak effect on ME180 cells and negative shRNA had no effect at all. It therefore appears that, in human cervical tumours, synergistically silencing DAF and MCP may be more effectively inhibit complement-mediated cytotoxicity.

The expression of DAF and MCP were significantly increased in human cervical cancer tissue. This finding suggested that DAF and MCP may play important roles in the survival of the human cervical cancer cells. According to our study (Figure 4A, B, and 4C), Cooperative inhibition the expression of DAF and MCP genes might decrease viability, migration and proliferation of ME180 cells. DAF and MCP may protect tumor from accidental injury by activated complement, also confer resistance on cancer cells. It seemed that DAF and MCP promotes cell viability, migraton and proliferation, even though the exact mechanism should be studied further. In the future trial we should provide a detailed analysis of complement regulatory protein expression on HPV infected and not infected human cervical cancer cells. We should also examine HPV infected and not infected premalignant cervical lesions and primary cervical squamous carcinomas to determine whether changes in the expression of these proteins are associated with the development of cervical disease.

## Conclusion

In conclusion, we were able to identify potent shRNA for efficient down-regulation of DAF and MCP expression on tumour cells. Knock-down of both surface-regulators clearly sensitized tumour cells to complement attack. This has been proved by analysis of complement-mediated cytotoxicity and also by investigation of C3b-deposition. The present data indicated that synergistical silencing DAF and MCP significantly decreased the human cervical cancer cell viability, migraton and proliferation. These data indicated that combined DAF and MCP shRNA described in this study may offer an additional alternative to improve the efficacy of antibody-and complement-based cancer immunotherapy in the future.

## Competing interests

The authors declare that they have no competing interests. The authors alone are responsible for the content and writing of the paper.

## Authors' contributions

LJG and SYG conceived of the study, analyzed data and wrote the manuscript. YQC, PQG, YJS and HG planned experiments and assisted in writing the manuscript. YL and CC analyzed the data. All authors have read and approved the final manuscript.

## Pre-publication history

The pre-publication history for this paper can be accessed here:

http://www.biomedcentral.com/1471-2407/9/384/prepub

## Supplementary Material

Additional file 1 **The expression of DAF and MCP levels**. Relative DAF and MCP expression levels were shown in four type of human cervical cancer cell line in this study. The level of DAF and MCP in ME180 was the maximal expression. Data are shown from one representative experiment (of three). Click here for file
