# Supplementary figures and images for: Cooperation of decay-accelerating factor and membrane cofactor protein in regulating survival of human cervical cancer cells
Source: BMC Cancer. 2009 Oct 30;9:384. doi: 10.1186/1471-2407-9-384 (PMC2774863; doi:10.1186/1471-2407-9-384)

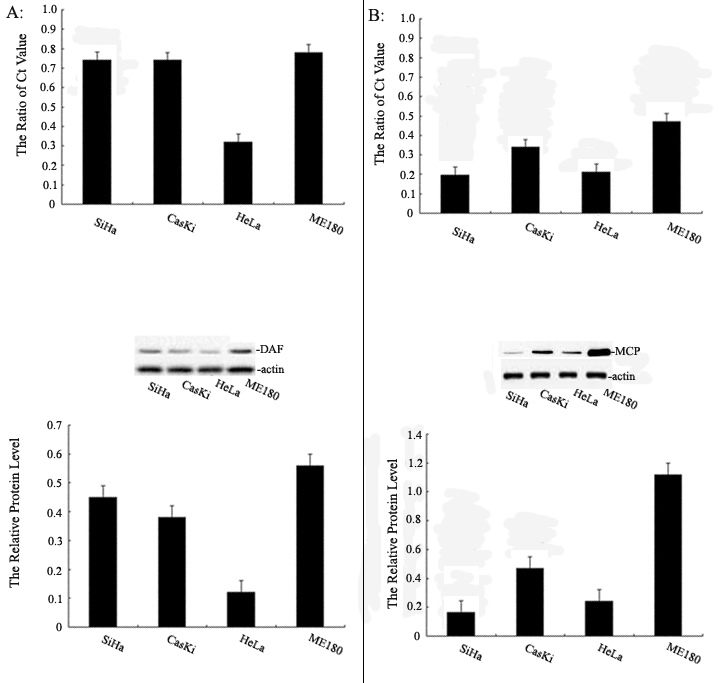

Supplement: Additional file 1 — The expression of DAF and MCP levels. Relative DAF and MCP expression levels were shown in four type of human cervical cancer cell line in this study. The level of DAF and MCP in ME180 was the maximal expression. Data are shown from one representative experiment (of three). [file 1471-2407-9-384-S1.jpeg]
